# Use of Secondary Reflectors for Enhanced ESWT Treatment of the Penis

**DOI:** 10.3390/biomedicines13081967

**Published:** 2025-08-13

**Authors:** Hannah Janout, Jonas Flatscher, Stephan M. Winkler, Paul Slezak, Cyrill Slezak

**Affiliations:** 1 Bioinformatics Research Group, University of Applied Sciences Upper Austria, 4232 Hagenberg, Austria; 2 Ludwig Boltzmann Institute for Traumatology the Research Center in Cooperation with the AUVA, 1200 Vienna, Austria; 3 Department of Computer Science, Johannes Kepler University, 4040 Linz, Austria; 4 Department of Physics, Utah Valley University, Orem, UT 84058, USA

**Keywords:** erectile dysfunction, shockwave therapy, shockwave simulation, evolutionary computation, optimization

## Abstract

**Background**: This study aimed to optimize low-intensity extracorporeal shockwave therapy (Li-ESWT) for the treatment of penile indications through the addition of a secondary reflector. The therapeutic potential of Li-ESWT is well-established, but its efficiency is limited by uncontrolled wave propagation and reflection resulting in regions of increased tensile pressures. The objective is to manage and reduce high tensile pressure and enhance treatment efficacy by reflecting applied shockwaves back into the treatment zone using a novel reflector design. **Methods**: A comprehensive investigation, including numerical modeling and phantom measurements, exploring a range of improvements to traditional shockwave application by reflecting applied therapeutic shockwaves back into the treatment zone. Computational optimization was employed to identify the most suitable secondary reflector shape for potential future clinical use. Subsequent hydrophone phantom reference measurements were extended to volumetric fields using 3D simulations. **Results**: Traditional treatment resulted in high tensile pressures in the treatment zone, which was mitigated by introducing an impedance-matched layer (IML) while preserving the initial shockwave’s therapeutic function. The addition of the secondary reflector enabled controlled refocusing of the therapeutic shockwave back into the initial focal zone, thus either increasing the treatment volume or achieving a rapid secondary application. Choice of the reflector’s impedance allowed for the secondary refocusing of either a tensile or positive pressure wave. **Conclusions**: The combined modifications of employing an IML and secondary reflector eliminate uncontrolled tensile waves and reflections, provide better control over consecutive reflections, and enable repeated shockwave signals with a single applicator shot, potentially reducing the number of required shots per session.

## 1. Introduction

Erectile dysfunction (ED) is the difficulty or complete inability to obtain or maintain an erection strong enough for sexual intercourse [1,2,3]. It is the most common sex problem reported by men, with an estimated 322 million men affected by 2025 [4,5]. ED can present as a symptom of various underlying problems, such as advanced age, poor vascularization, diabetes, medication, depression, or stress, and remains one of the most commonly diagnosed penile indications [4,5,6,7,8,9].

The most common treatments for ED consist of oral phosphodiesterase type 5 inhibitors (PDE5i), the most commonly known one being Viagra, or intracavernosal injections of vasodilating agents. The treatment is taken on demand before sexual activity and only lasts for several hours, affecting the spontaneity of the affected person. Although these treatments are very effective and safe for patients, none treat the underlying pathophysiology of the erectile mechanism [4,5,6,10,11,12,13,14,15]. Only in a small fraction of the affected population do established treatments, such as a change in lifestyle, drug regimen, or microvascular surgery, offer the possibility of regaining spontaneous erectile function [11]. Additionally, ED not only seriously affects a patient’s quality of life, but may also be an early symptom of cardiovascular disease [13].

Extracorporeal shockwave therapy (ESWT) is a non-invasive treatment that uses high-energy, short-duration acoustic pulses to promote healing and tissue repair. Initially developed for the destruction of kidney stones, lower-energy ESWT has since become an increasingly popular treatment method for a wide range of indications, with proven success particularly in soft tissue regeneration and pain relief, and offers a non-invasive modality in the medical field for the treatment of various musculoskeletal disorders [16,17].

The therapeutic effects of low-intensity extracorporeal shockwave therapy (Li-ESWT) are attributed to its ability to activate cellular pathways associated with regeneration, an increase in the expression of local growth factors, enhance endothelial function, promote angiogenesis, and possibly even contribute to the regeneration of nerve fibers [18,19,20]. In 2010, Vardi et al. [21] reported the first application of Li-ESWT for treating ED in male patients. The study found significant improvements in erectile function, including increased IIEF-ED domain scores, duration of erection, penile rigidity, and endothelial function [21]. Since then, after years of clinical observation, Li-ESWT established itself as an effective treatment method for ED and, in particular, has shown promise as a treatment for vasculogenic ED, representing an approach that offers the potential for a cure [11,13,22,23,24].

Despite these apparent successes, there is no standardization for ED treatment through Li-ESWT, and a wide range of treatment protocols have been reported. They differ in aspects such as the number of treatment sessions, delivered shocks, energy flux density, device type, and device positioning [25,26].

ESWT is currently provided utilizing three kinds of generating technologies to create therapeutic shockwaves: electrohydraulic, electromagnetic, and piezoelectric. This work concentrated on weakly focused, parabolic electrohydraulic (EH) shockwave devices. Its waveform naturally shows a high compressive peak and a comparable low tensile peak pressure even at lower energies, making it ideal for the separate investigation of both positive and negative pressures. EH devices generate a therapeutic shockwave through high voltage discharge across two electrodes [17]. A spark-induced plasma bubble rapidly expands until a shockwave separates. The applicator’s reflector then focuses the spherically spreading shockwave in the applicator towards a designated focal zone outside the applicator’s head and into the treatment zone. The primary unencumbered shockwave continues to precede radially while the reflector-incident part is redirected into the focal volume [17]. The pressure waveforms generated by EH devices are stochastic due to variations in the intensity and location of the spark between the two electrodes, which creates variations in the resulting shockwaves’ shape and energy.

As shockwaves propagate inside the body, they are reflected and phase-inverted when they encounter an air interface, i.e., positive pressures become negative and vice versa. During therapy, inversion occurs when the shockwaves continue traveling through the treatment zone, still carrying most of their energy, and subsequently coming into contact with the air interface. Due to different placement angles of the applicator, variations in patient geometry, and variations in handling of the penis by the therapist since most therapy sessions are performed handheld, these inverting reflections may happen at different times and intensities throughout a therapy session, resulting in side effects associated with cavitation formation, such as bruising of the area [17,27,28,29].

**Figure 1:**
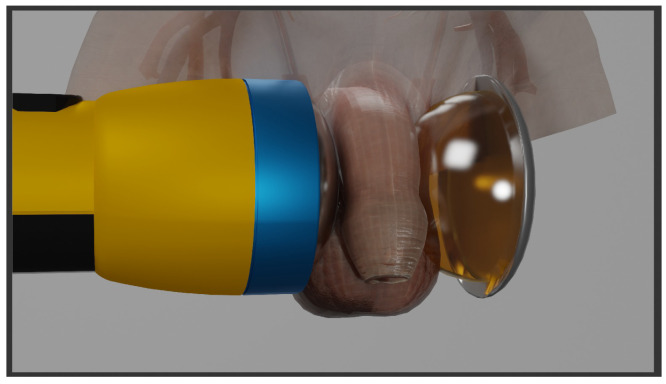
A graphical depiction of the therapy setup with the applicator applying shockwaves from the left. An impedance-matched layer (gelatinous dome) with an attached symmetry-matched secondary reflector (silver shell on the far right) are positioned inline, on the opposite side.

This study addresses variations in current ED treatment setups, particularly the impact of introducing a secondary reflector and an impedance-matched layer (IML) to enhance the traditional treatment setup (see Figure 1). This investigation will focus (1) on the mitigation of tensile pressures by means of an IML and (2) the ability to refocus the dispersing energy of the shockwave after it has passed through the treatment area once by introduction of a secondary reflector. First, we designed an optimized 3D symmetry-matched secondary reflector by using an evolution strategy. Second, the thus designed reflector was evaluated in experimental phantom-based measurements before further exploring the volumetric properties by means of numerical simulations of the ESWT application.

## 2. Materials and Methods

### 2.1. Overview

**Figure 2:**
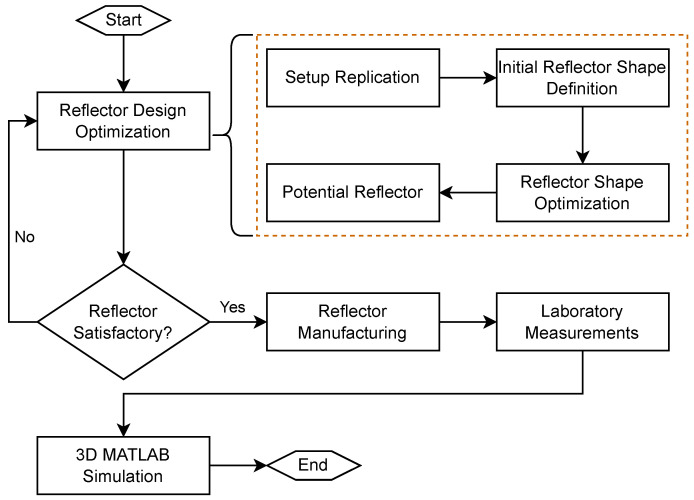
Flowchart depicting the workflow of the presented study. The first step consists of finding the optimal shape for the secondary reflector through a tailored evolution strategy with genetic programming, as detailed in the dashed, orange box. Once a satisfactory reflector design was achieved, the obtained reflector was evaluated in the laboratory. Lastly, 3D simulations provided further insights into the volumetric pressure distributions.

Our method’s primary focus is the investigation of a possible recycling of shockwaves past their initial pass through the focal zone, by the removal and controlled refocusing of the initial shockwave back into the therapy zone. The study’s workflow steps presented in this paper are visualized in Figure 2: reflector design optimization, reflector manufacturing, laboratory measurements, and 3D simulation. The in-set orange box provides a more detailed view of the optimization steps to find the best-suited reflector shape.

For the reflector design optimization, we numerically evaluate a traditional treatment setup using a weakly focused, parabolic electrohydraulic shockwave applicator to determine the best-suited reflector shape and convert the task into a minimization problem. The optimization is performed in two phases: first, a fixed positioning of the secondary reflector is assumed to determine its maximal reflection ability, and second, positional variations of the reflector are introduced to mimic handling during a therapy session in order to obtain a final stable reflector design.

Following manufacture, the optimized reflectors were evaluated in the treatment of a gelatin penis phantom in an experimental lab setup to mimic a therapy application. This allowed point-wise in situ pressure measurements to validate the optimized design and functionality.

Lastly, a custom 3D simulation of the entire setup allowed us to gain insight into the various pressure distribution, volume, and intensity over the whole penis, which are not observable in point-wise in situ pressure measurements. This enabled a holistic side-by-side comparison of various experimental configurations for potential future clinical use.

### 2.2. Applicator Replication

For the investigation presented here, the Orthogold100 using the OP155 applicator (MTS Medial, Konstanz, Germany) was chosen, as they are frequently used in clinics worldwide [30]. This applicator device has a relatively large focal field and poses the largest optimization challenge due to its weakly focused focal zone and stochastic generating nature.

The first step of the reflector design was creating a numerical 2D water bath setup [31] design for shape optimization, removing influences from media interfaces, and guaranteeing a clean sound field for traveling shockwaves. During shape optimization, a planar reduction of the applicator’s rotational symmetric primary reflectors along with a point source was positioned opposite the to-be-designed secondary reflector as depicted in Figure 1.

### 2.3. Reflector Design and Optimization

The heart of the optimization workflow consisted of an evolution strategy (ES) tailored to design and modify the reflector shape. An ES is a computational optimization strategy that draws inspiration from the biological principle of evolution and natural selection [32,33]. It works with a population of solution candidates (i.e., possible solutions) for a given problem. Furthermore, each solution contains a quality metric that informs its ability to solve the problem and allows for precise ranking and selection of individual solutions in the population. Depending on the problem to be solved, the quality metric is either minimized or maximized. A percentage of the population is transferred to the next generation and used to initialize and modify, also called mutate, new solution candidates before evaluating their quality metric, slowly searching for the best possible solution to the given problem. Thus, the evolution strategy can be summarized in three basic steps: initialization, mutation, and evaluation [32,33].

In this study, the problem was defined as finding the reflector shape that best refocuses shockwaves past their initial pass back into the shockwave applicator’s original focal zone. A single reflector shape represented a possible solution to this problem and was defined as the solution candidate; its quality metric directly corresponding to the reflector’s ability to reflect and refocus shockwaves into the shockwave applicator’s original focal zone. As the optimization of the secondary reflector is defined as a minimization problem of the quality metric (distance to the original focal zone), it is to be minimized.

During the reflector optimization, the propagation of a shockwave pulse was modeled and represented through rays in order to simplify, and, thus, enable a more flexible optimization. These simplifications allow a more dynamic and multi-parametric optimization, especially since the final reflector designs were subsequently validated.

#### 2.3.1. Solution Candidate Initialization

For the reflector shape optimization, a solution candidate was initialized with various parameters, the most important of which are as follows.

- **Geometry**: The elliptical secondary reflector is defined by its major- and minor-axis (*a* and *b*, respectively) at the fixed position (h,k).
- **Quality**: The quality of the candidate, describing the reflector’s ability to refocus shockwave rays into the shockwave applicator’s original focal zone. Calculation of the quality is defined in Section 2.3.3.
- **Penalty**: A penalty for violating constraints, such as minimum reflector size, which constricts handling of the secondary reflector. Further explanation of the penalty can be found in Section 2.3.3.

The initial ellipse parameters were set randomly for each candidate, while all other parameters were set to predefined constants.

#### 2.3.2. Solution Candidate Mutation

The mutation step of an ES modifies the parameters of a solution candidate to create a new solution for the problem. For the solution candidate mutation of the secondary reflector, the ellipse’s axes were modified by decreasing or increasing their value, thus altering the shape of the secondary reflector. This enables the ES to explore the problem search space and possibly find a better solution to the problem. The mutation is defined as

(1) $$\begin{array}{c} a_{new}=a_{old}+r_{a}\;\delta \\ b_{new}=b_{old}+r_{b}\;\delta \end{array}$$

where ***a*** and ***b*** define the ellipse’s axes, ***r*** represents a random value between −1 and 1 drawn from a uniform distribution, and **δ** describes the mutation coefficient. The random variable *r* gives the mutation’s direction, increasing or decreasing the previous value. The mutation coefficient δ determines the extent of mutations within the population and adapts dynamically between generations. High δ values lead to significant mutations, expanding the search; in contrast, low δ values result in minor mutations, concentrating the search. The value is adjusted using the $\frac{1}{5}$ rule, which increases or decreases δ based on the success rate of mutations [34]; while δ is rather high in the beginning, it decreases as the algorithm reaches later stages of the evolution.

#### 2.3.3. Solution Candidate Evaluation

The reflector’s primary attribute is its ability to refocus shockwaves after passing the treatment zone back towards the applicator’s focal zone. Each solution candidate is evaluated based on its ability to fulfill this criterion. The better a solution candidate’s ability to reflect and refocus the shockwaves, the better its quality.

**Figure 3:**
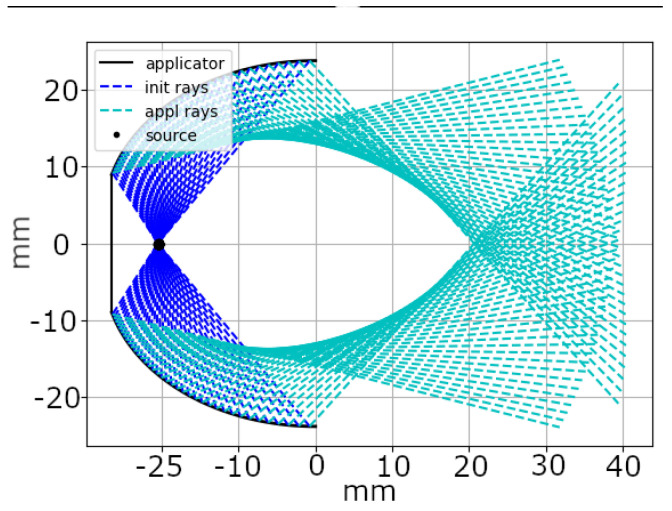
Two-dimensional visualization of the applicator, including the generated shockwave’s origin position and the subsequent computed propagation rays. Initial rays outgoing from the origin are marked in dark blue, and change to cyan after their initial reflection. The applicator’s soft focus can easily be spotted by the convergence and locally increased density of rays on the right.

For this evaluation, we computed the travel path of the emitted shockwaves for each candidate solution. This process involved following the rays from their origin within the applicator’s spark gap, known as the origin position, to the point of impact on the reflector and the subsequent reflection. Figure 3 illustrates this path. Without interference, rays originating from the applicator’s origin position (X,Y) traveled in a direction represented by the vector $\left(d_{x},d_{y}\right)$, shown as dark blue rays in the figure. Upon intersecting the applicator’s reflector at position $\left(X_{i},Y_{i}\right)$, these rays were reflected, changing their direction to $\left(d_{x,refl},d_{y,refl}\right)$, which was depicted as cyan rays.

Using Snell’s law [35] to compute the rays’ reflection behavior, determining each emitted ray’s travel path becomes possible. While Snell’s law is not entirely applicable to non-linear shockwaves, it provided a sufficient approximation of the reflection behavior for the shape optimization.

The quality of a solution candidate was calculated as the average Euclidean distance between the focus point of the applicator $\left(X_{focus},Y_{focus}\right)$ and the reflected rays at position $X_{focus}$. Therefore, a candidate with a low distance value indicated a better solution as the average Euclidean distance is low, which indicated an enhanced ability to focus shockwaves. The exact formula for the computation of the distance and definition of quality for one position is given in Equation (2). *N* is defined by the total number of rays being reflected, while $\left(X_{focus},Y_{focus}\right)$ denotes the applicator’s focus point, and $\left(X_{refl},Y_{refl}\right)$ denotes the secondary reflector’s focus point.

(2) $$quality=\frac{1}{N}\sum_{i=1}^{N}\left|Y_{focus}-Y_{refl_{i}}\right|+penalty$$

Each solution candidate was penalized for deviations from permitted reflector shapes or behaviors during optimization. This penalty considered the minimum and maximum sizes for the semi-major and semi-minor axes, ensuring practical, easily handheld reflectors. It also accounted for the number of rays failing to meet the reflector. By examining the rays that missed the reflector, instances where a reflector only intercepts a small percentage of incoming rays could be disregarded, regardless of its possibly superior ability to refocus. We have analyzed the concurrent time of arrival (lengths of the rays), which is essential for the re-formation of a shockwave, in detail for different setups, finding a low impact on the quality of a solution candidate. We have thus decided to omit the concurrent arrival penalty from the optimization in favor of increased stability and efficiency of the evaluation process. The penalty guaranteed that the reflector optimally refocused the most rays to maximize pressure in the therapy zone and minimized interactions between shockwave rays and the surrounding air in the treatment zone.

The reflector position was kept constant in the first steps of the reflector shape optimization, which was also subsequently experimentally validated. This mimics the application of the secondary reflector as a fixed apparatus connected to the applicator head, and allows for determining the best achievable quality value for the reflector. In later steps, the reflector position was varied to determine the reflector’s robustness towards possible positional changes during therapy and guarantee consistent performance of the reflector even during changing therapy scenarios. This variation consisted of increasing or decreasing the distance between the secondary reflector and the applicator device, and in the up and down movement of the secondary reflector. This optimization step computed the quality separately for each new reflector position and ultimately averaged to achieve the final quality value.

The three main steps, initialization, mutation, and evaluation, are repeated for a new population of solution candidates during each generation, each generation being based on the solution candidates of the previous one. The algorithm terminated upon the criteria of stagnating quality or maximum generations. Either way, the evolution strategy returned a population of reflector parameter combinations well-suited for refocusing the shockwaves back toward the applicator’s original focus. The reflectors of the best solution candidates were used for further evaluations.

### 2.4. Phantom Measurements

**Figure 4:**
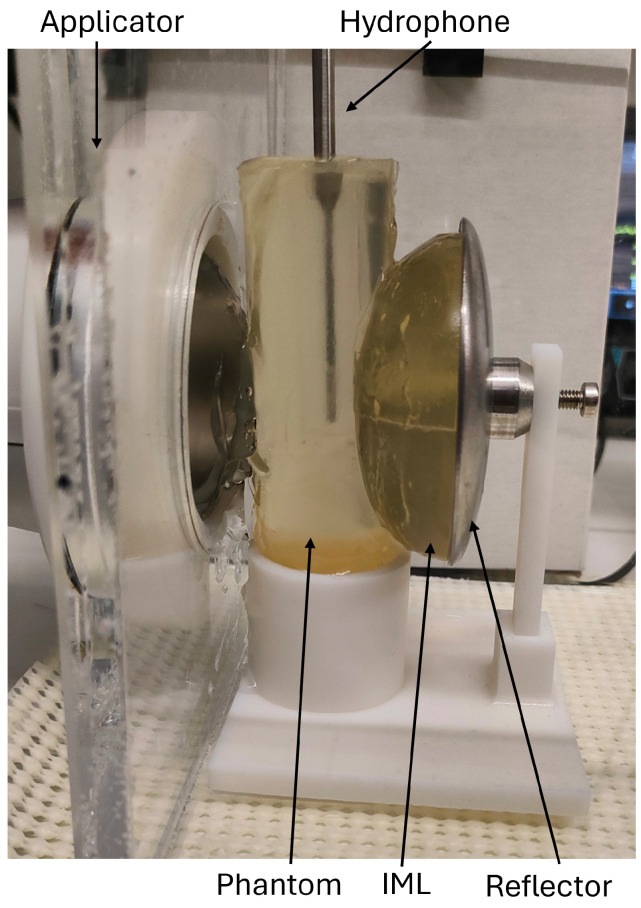
Visualization of the laboratory setup including the applicator, penis phantom, hydrophone, IML, and designed secondary reflector.

To experimentally evaluate the reflector’s functionality, a phantom therapy application was set up (see Figure 4). A suitable [36,37] gelatin penis phantom and an IML were produced by pouring gelatin into a 3D-printed mold. The gelatin concentration was chosen to be 15% to mimic the acoustic properties of the human body and provide mechanical stability for the test setup [38]. The penis phantom was axially hollowed, creating an 11 mm diameter cylindrical hole, and filled with water to assure proper acoustic coupling with the hydrophone. Furthermore, a 3D-printed holder was designed to align the penis phantom centered in the focal zone and relative to the different reflectors’ positions. A needle hydrophone (Müller Instruments, Germany, Oberursel) was used to measure the shockwave signal in the center of the phantom, using the OP155 applicator head at an energy setting of 0.18 mJ/mm^2^;. As the pressure wave needs to be recorded both in the incident and reflected pathways, the hydrophone was positioned vertically—perpendicular to the propagation axis. While the sensor is calibrated only for direct incident pressure, the signal attenuation for off-axis use can be determined based on manufacture specifications [39] to −7 dB at 90°, making it possible to estimate the original pressure, quantify the ratio of the incident to the reflected shockwave, and thus determine the effectiveness of the designed reflectors.

The use of two hydrophones was considered, pointing in opposite directions—this setup would have required the sensors to be positioned at a steep incident angle due to the applicator’s and the reflector’s geometry, providing no improvement while increasing the complexity.

Two reflectors with the same surface geometry according to the optimization were fabricated for testing: one made out of high-alloy stainless steel (X5CrNi18-10) and the second one created to produce an effective air reflection with minimal structural support in the form of a 3D-printed honeycomb pattern (Form 2, Formlabs white resin v4). A generous amount of ultrasound gel was used to couple the interfaces and remove air inclusions at component connections.

### 2.5. Reflector Simulation

The same secondary reflector design as in the laboratory measurements was integrated into a 3D simulation in MATLAB, version 2023. The computational simulations used the k-wave [40] toolbox, designed to simulate and reconstruct photoacoustic and ultrasound wave fields. The simulations were conducted with a spatial grid spacing of 0.35 mm, and the simulated 3D volume was 87.2 mm × 63 mm × 63 mm with a perfectly matched layer (PML) thickness of 20 grid points, or 7 mm, added along each border.

The simulated applicator reflector was again taken after the OP155 applicator head, and the penis was represented by a cylinder with a radius of 14.7 mm based on the study of Vaele et al. [41] of average flaccid penis dimensions. The representative homogeneous acoustic tissue parameters of the penis are taken from the Foundation for Research on Information Technologies in Society (IT’IS Foundation) [38].

The IML between the penis and the reflector was chosen to be a 14% saline solution to match the impedance of the penis. Maximum and minimum pressures were recorded for each voxel throughout the simulation. The reflector was added as an ellipsoidal shell on the opposite side of the penis, with the semi-minor and semi-major axes taken from the optimized reflector design. The reflector material was either steel or air medium, and the surrounding medium was set to air. Furthermore, virtual sensors were added to record the pressure waves in critical positions during each step of the therapy session.

## 3. Results

### 3.1. Reflector Design and Optimization

The first results for the evolutionary optimization were conducted with a fixed center position for the reflector. In later experiments, the central position was varied for each solution candidate, mimicking movement during a therapy session to test the secondary reflector’s shape for robustness towards movement.

The first population of the evolutionary optimization is created randomly and may vary significantly between optimization runs. Hence, the optimization workflow was executed 100 times, with the best results being saved for further evaluation. Repeated optimization allows for the elimination of lucky or bad guesses made by the algorithm. This repetition increases the algorithm’s chances of finding a globally optimal reflector shape instead of getting stuck in a local optimum. Furthermore, repeated computation shows the algorithm’s preference, or lack thereof, regarding the reflector’s shape and the algorithm’s robustness.

**Figure 5:**
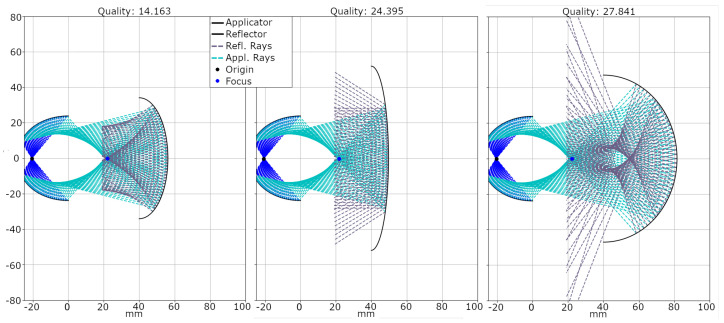
Depiction of three solution candidates of the ES and their ray distribution. The number on top indicates their evaluated performance quality. The source point of the shockwave is highlighted as a black dot in the applicator head, and a blue dot represents the reference point in the focal zone used for computing the quality. The zero position corresponds to the applicator’s aperture.

Figure 5 shows the results of different optimization runs with a fixed center position and their corresponding quality indicated above. The plots in the figure are ordered based on their quality, with the best located on the left and the worst on the right.

Both optimizations, fixed and varied positions, resulted in a shallow, ellipsoid shape for the secondary reflector, which effectively focuses incoming shockwaves back towards the original focal zone. Very deep and broad reflector shapes reflect the incoming shockwave, but tended to create the focal zone too close, sometimes inside the reflector itself, and formed multiple focal zones. On the contrary, extremely shallow reflectors tended to disperse the shockwave instead of focusing. In some cases, the shape of the candidate was outside the allowed guidelines, which resulted in a high penalty for the candidate and, consequently, a poor quality.

To further analyze the robustness of the results of the evolutionary optimization under mobile positioning, we conducted an additional 50 ES runs to compare stationary and variably positioned reflectors. The best performing solution candidates of the ES runs with a fixed center had a quality of 14.16, with only the third decimal differing slightly, with the worst performance reaching 49.57. The average best quality was 15.33 (±1.73), the low standard deviation and low mean value highlighting the ES’s robustness towards reliably finding the best possible reflector shape. For the optimization involving movement, the best achieved quality was at 24.76, with the worst candidate being at 67.08. The average best quality for these optimization runs was 29.36 (±4.92). The larger standard deviation and mean value indicate how the inclusion of reflector movement decreases the algorithm’s robustness, while the ES was still able to return valid reflector shapes reliably.

### 3.2. Phantom Measurements

We experimentally measured the shockwave signals for four treatment configurations: (a) a traditional ED therapy setup, (b) an extended setup including an IML-based absorber, (c) an effective air reflector using a highly porous 3D-printed honeycomb structure to provide stability to the IML, and (d) a steel reflector (Figure 4).

**Figure 6:**
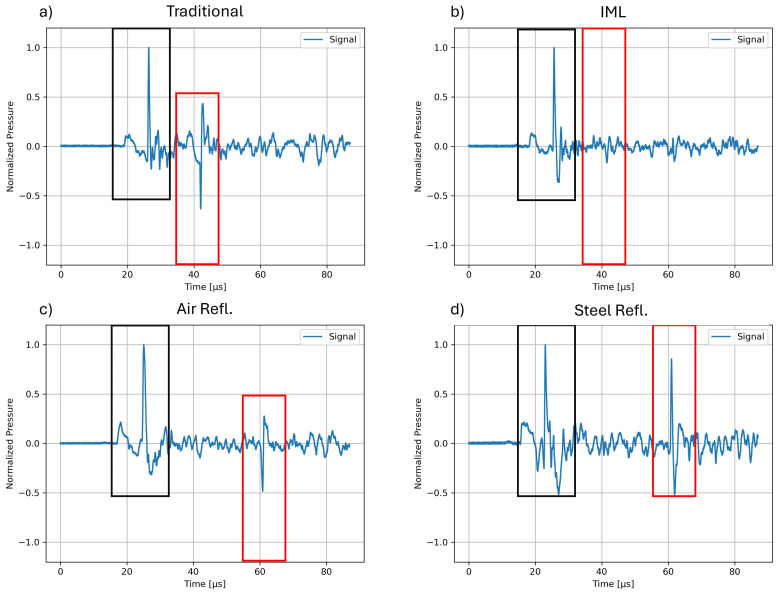
Measured pressure waveforms in four experimental configurations: (**a**) a traditional ED therapy setup, (**b**) includes the IML without back-reflection only, and final configurations including a secondary reflector made of (**c**) air and (**d**) steel. The primary and focused shockwave signals are denoted in black rectangles, while additional refocused reflections are denoted in red rectangles. Signals were normalized to the peak pressure of the primary peak to ensure consistent evaluation and comparison.

Figure 6 shows representatives of the recorded pressure waveform within the penis phantom’s center for each configuration. The black rectangle in each image highlights the EH shockwave device applicator’s focused shockwave and primary wave (small preceding peak). Red rectangles mark further reflections created independently from the applicator. Since EH devices create stochastically varied pressure amplitudes due to the variability of the spark discharge, waveforms were normalized to their respective initial peak pressure to allow for a direct comparison.

In the traditional ED therapy setup (Figure 6a), the original, primary wave and focused shockwave of the EH device is appended by an inverted signal only 10 μs later. This is due to the shockwave’s phase-inverting reflection when encountering the air surrounding the phantom. The perfect cylindrical form of the model refocuses the wave, resulting in a significant tensile pressure and small positive wave components of a secondary pulse (framed by a red rectangle).

The second configuration (Figure 6b) incorporates an IML-based absorber, a layer of material with similar acoustic properties as the penile tissue, which was placed opposite the applicator. The role of the IML is to effectuate an unimpeded propagation of the shockwave out of the treatment zone by effectively displacing the air interface at the back of the penis well into the IML, leaving the tissue unaffected. Any secondary reflections at the far end of the IML will be random due to the lack of a cohesive shape of the interface. This effectively creates an acoustical absorber such that little and not focused pressure waves return into the penis.

Next, a secondary air reflector with a honeycomb pattern was introduced and placed at the back of the IML (Figure 6c). This terminates the IML and its air interface, giving it a concrete shape and position, resulting in a controlled reflection and inversion of the initial shockwave. The resulting, focused tensile pressure wave measured in the phantom’s center (indicated by the red box) is less intense than in the traditional setup but still more intense than the tensile pressure of the initial shockwave. At the same time, the resulting waveform has only a minute positive pressure component and is delayed in its secondary pass through the treatment zone by 30 μs.

In the final configuration (Figure 6d), a steel reflector was considered instead. Unlike the air reflector, steel reflects the shockwave without causing phase inversion, thereby maintaining the pressure in its original phase. At the same time, the introduction of the IML mitigates potentially heightened tensile pressure. As highlighted by the red rectangle, the returned signal from the steel reflector closely matches the proportions of the initial shockwave. Importantly, the reflected pulse retains the characteristic wave form of the initial shockwave, indicating effective reflection and refocusing by the steel surface. This results in two shockwave pulses at the center of the penis phantom from a single applicator shot, effectively doubling the therapeutic application.

### 3.3. Reflector Simulation

Direct measurements of the previous sections are unfortunately unable to provide a volumetric evaluation of the shockwave application. To this end, we replicated the laboratory configurations in a 3D non-linear acoustic simulation based on the k-wave toolbox [40]. The same four configurations as in the laboratory measurements were simulated.

**Figure 7:**
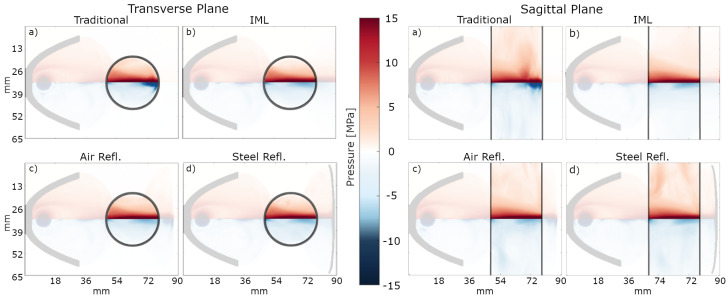
Depiction of each reflector’s axis-symmetric peak positive and tensile pressure distributions indicated by the respective color gradient in both the transverse and sagittal planes. For easier visualization, the opacity value of the area outside the penis was reduced. Configurations depict (**a**) the traditional setup, (**b**) the IML absorber, (**c**) the air reflector, and (**d**) the steel reflector.

Figure 7 shows the maximum (red) and minimum (blue) axis-symmetric peak pressure recorded in the transverse and sagittal plane of the simulation for each configuration. The visualized value range is −15 MPa to 15 MPa, to equalize the color scheme for the individual simulation result; values above or below this range are capped for easier visualization. In addition, the opacity of the area outside of the penis phantom has been reduced to highlight the region of interest.

For each configuration, the peak positive pressure is quite similar in shape and intensity and dominated by the initial pass of the shockwave. In contrast, the traditional application results in high-intensity tensile pressure at the rear of the numerical phantom, extending deep into the tissue. This high-intensity tensile pressure region reaches peak values of up to −24 MPa, 2.4 times higher than the peak tensile pressure in the other configurations. With the introduction of the IML absorber, the high-intensity tensile pressure of the traditional configuration is eliminated inside the penis and replaced by evenly distributed low-tensile pressure, as the shockwave can propagate through the medium without reflection. While the addition of the IML leaves the peak positive pressure distribution almost completely unaffected, the extreme tensile peak pressure appearing during a traditional ED configuration is effectively displaced outside the phantom. The addition of the respective reflectors only slightly impacts the peak pressure distributions, with each showing only a slight increase in regions of peak positive (steel) and peak negative (air) over the IML alone. This can be attributed to the dominance of the initial shockwave pass as observed in the experimental result, and secondary passes are superimposed as the secondary reflector was re-aligned with the primary focal zone of the applicator.

**Figure 8:**
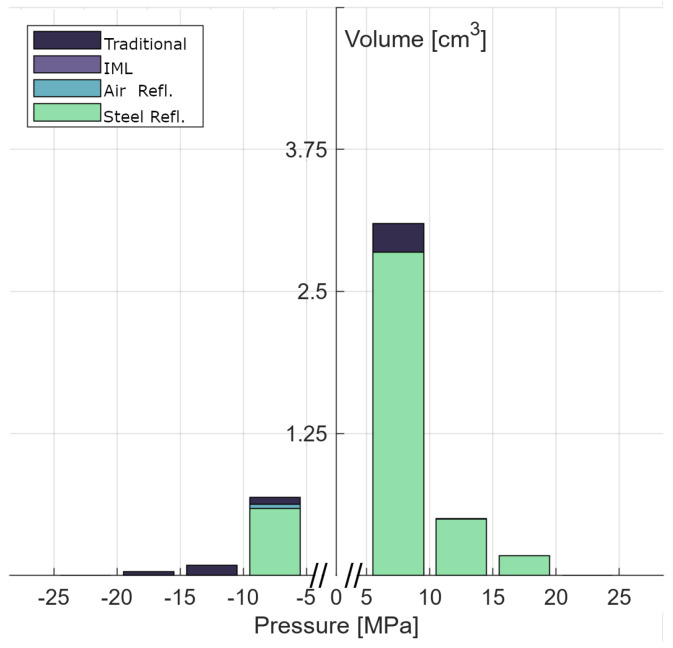
The peak pressure distributions of each configuration are displayed as overlapping bars. Peak pressure values between −5 and 5 MPa were omitted for better visualization. The positive pressure distributions are found to be similar for all configurations, while the peak tensile pressure distribution of the traditional and air reflector setups differs significantly from the rest.

In order to obtain a more quantitative assessment, Figure 8 quantifies the treated volume in cm^3^ by peak pressure. The smallest of peak pressure values, between −5 MPa and 5 MPa, were omitted to focus purely on the focal zone and exclude areas far removed from the focal zone due to the comparably large volume of the phantom. A Kruskal–Wallis ANOVA finds no statistical difference (H(3) = 1.00, *p* = 0.802) in the distribution of volumes of positive peak pressures at all energy levels. Between 5 and 10 MPa, only the traditional configuration displays a slightly larger volume of 3.1 cm^3^, while IML, air reflector, and steel reflector reach a volume of 2.8 cm^3^.

In contrast, we see a statistically significant effect (H(3) = 6970.49, *p* < 0.001) in the distribution of volumes of tensile peak pressures. Dunnett’s multiple comparison test showed significant differences in peak tensile pressure between the traditional and all other configurations (*p* < 0.001). This is very apparent in the graph, as pressures below −10 MPa are only found in the traditional setup. We do, however, also observe a smaller, yet significant difference (*p* < 0.001) for the air reflector in the lowest pressure range.

An undisturbed shockwave pulse during therapy exhibits a single focused shockwave peak followed by an elongated tensile pressure phase, as seen in the measurement in Figure 6b. Any additional subsequent peaks in a local pressure reading originate from additional reflections and refocusing. By evaluating the number of peaks per simulated configuration, we can determine the number of shockfront passes (i.e., pulses) occurring at a specific location.

**Figure 9:**
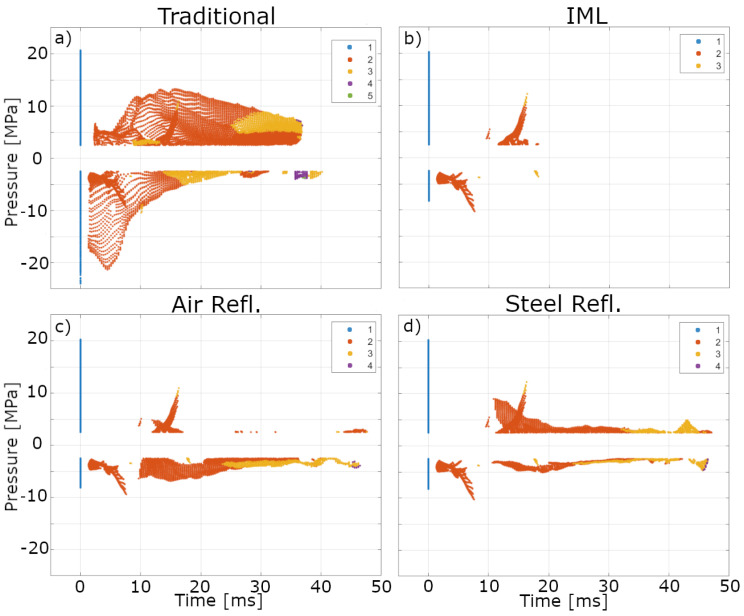
Scatter plots displaying the simulated peak pressure of a reflected shockwave pulse as a function of the time past the initial, at a given location for the four experimental configurations. The coloration indicates the enumeration of a subsequent peak, with the initial peaks seen in blue at time 0. Image (**a**) depicts the traditional setup, (**b**) includes the IML, (**c**) includes the IML and air reflector, and (**d**) shows the IML and steel reflector.

Figure 9 shows the intensity of each enumerated (identified by color) subsequent pass and their respective arrival times after the initial pass-through. Only passes with a minimal peak intensity of 2.5 MPa or −2.5 MPa were considered with a minimum peak prominence of 1.5 and minimal distance between peaks of 1.298 μs. Positive and tensile peaks were determined independently of each other.

Figure 9a depicts a largely different outcome for the traditional application compared to the three experimental ones. This configuration results in multiple reflections (up to five peaks) stemming from repeated reflections at the air interfaces, alternating between in-phase and phase-inverted pulses. We also note overall areas of the penis phantom to experience significantly higher pressure exposure than others.

Through the introduction of an impedance-matched layer (Figure 9b), the majority of recurring peaks disappear. Only a small portion of secondary and tertiary peaks remain, which stem from reflections within the applicator; these reflections are observable in all four configurations and establish a key reference for analysis.

Results for the reflector with an air medium (Figure 9c) show the expected increase in tensile peak pressures over the IML absorber configuration, with a maximum of four peaks for the respective locations. These refocused reflections are similar to the tensile pressure of the original focused shockwave, with no significant outliers observed. In contrast, the steel reflector (Figure 9d) exhibits significant additional positive peak pressure pulses over the IML absorber setup, while the additional, albeit still present, much weaker tensile peaks remain.

Among the results, the traditional application stands out. Although the same features of the IML absorber are still present, additional, very large structures remain. The reason behind the large amount and high intensity of the reflections in the traditional ED therapy setup can be shown to result from the perfectly cylindrical shape of the penis phantom. This ideal geometry facilitates refocusing of reflected wave pulses, which only have the applicator’s comparatively small contact area to escape the phantom. For verification, a second penis phantom incorporating random surface noise by alternating elliptical cross sections (on average varying the radius by 8.9%) was created. These surface irregularities remove the perfect cylindrical symmetry, thus reducing the intensity of the secondary tensile pressure peaks of the traditional configurations by a factor of two. There is simultaneously a reduction in the positive peak pressure, while quaternary and quinary peaks disappeared entirely. Only minute, insignificant differences were observed using this irregular phantom in the other configurations including the IML and reflectors (see Appendix A), providing clear evidence that the overestimation of the traditional application results shown in Figure 9a are artifacts of the perfect geometry and not representative of what would be seen in a clinical treatment. Nonetheless, the peak tensile pressure in the traditional configuration remains at least a factor of two higher than in the other setups, indicating that geometric irregularities alone cannot fully mitigate the intense tensile pressures observed in the smooth phantom.

## 4. Discussion and Conclusions

There is currently no standardization or widely established treatment protocols for the Li-ESWT treatment of penile indications, resulting in wide variations in treatments depending on the therapist. Frequently, treatments are applied in a handheld fashion—with the penis in one hand and the device applicator in the other. Therapists have reported feeling shockwave pulses in their hands during this type of therapy. This phenomenon can be attributed to two factors: (1) By considering the acoustic coupling between the penis and the therapist’s hand, we can identify that in combination, the moisture build-up inside the examination gloves and liberal use of ultrasound gel creates an effective acoustic coupling, which enables the shockwave to propagate through the treatment zone and into the therapist’s hand. (2) Given the diameter of the penis and the extent of the applicator’s focal zone (as well as any applicable spacers), much, if not all, of the focal zone may be inside the therapist’s hand, creating the aforementioned sensation.

The combination of these two factors indicates an inappropriate shockwave application. For one, the hand takes on the effective role of the impedance-matched absorber layer in our investigation, and for two, the focal zone is likely misaligned. It is noteworthy, however, that the hand creates an effective IML that will remove much (depending on the coupling) of the accumulated tensile pressure regions in the penis. Combining this with uncontrolled reflections and phase inversions at the air–tissue interface, along with the absence of standardized treatment protocols, introduces substantial variability and uncertainty in Li-ESWT for erectile dysfunction. Differences in applicator placement angles, patient anatomy, and therapist handling can lead to reflection and inconsistent intensity throughout a therapy session. These factors may contribute to cavitation-related side effects, such as localized bruising [17,27,28,29]. The use of an IML minimizes the air interfaces, thereby significantly reducing the occurrence of intense tensile pressure regions within the tissue. This alone is already a substantial improvement to the therapy as it stabilizes the acoustic environment. Using a secondary steel reflector takes this a step further by enhancing shockwave delivery and increasing therapeutic efficiency.

When introducing the steel reflector, shockwaves from the applicator after their initial pass through the focal zone are reflected in-phase and refocused back into the treatment zone, re-exposing the tissue to a second pulse. This effectively creates twice as many shockwave pulses with a single shot within the focal zone, reducing the required number of pulses per therapy session and potentially increasing therapy efficiency. Both our experimental and simulated data support that in this application, there is no alteration of the initial shockwave pulse and that the secondary one is of comparable shape within the same, overlapping focal zone. Alternatively, the secondary reflector’s focal zone can be adjusted to target a different volume within the treatment zone from the applicator, thus increasing the effective focal zone. This aspect may be especially interesting to shockwave devices with relatively small focal zones, such as piezoelectric, as it increases the treatment volume with minimal effort.

In this study, we further explored a secondary reflector utilizing air as a reflector medium. While there may be limited clinical relevance for this phase-inverting reflector, it provides great potential for further research about the nature of mechanotransduction. Through controlled spatial separation of an initial shockwave and subsequent refocused tensile pressure wave, a direct comparison between therapeutic tissue repose under both positive and negative pressure can be systematically studied. The resultant wave pulse is, however, unable to fully recover the inverted shape of the positive pressure pulse due to underlying non-linearity effects and absorption of the highest spectral frequencies during refocusing. Especially for electrohydraulic applicators’ inherent shockfront in the pressure wave, maintaining much of the waveform of the initial application, the newly formed tensile wave can provide further insights into the effects of tensile forces on tissue and their role in regeneration.

These reflectors are cost-effective to produce, are reusable, and require no maintenance. Sterilization procedures for both the secondary reflector and the IML align with standard protocols used for the applicator, minimally impacting clinical practice.

The general applicability of evolution strategies to a wide range of optimization problems is well known. Using a specifically tailored quality metric, we were able to optimize an ellipsoidal secondary reflector shape for the refocusing of parabolic, weakly focusing electrohydraulic devices with softly focused focal zones. One key consideration in the optimization of shockwave reflectors is the high computational cost of non-linear propagation simulations. Even though it would be possible to divide the simulations into a universal pre- and individual post-reflection regime and thus only needing to simulate the waves after interacting with the secondary reflector, such an approach would only reduce the computational time by a factor of two while still requiring the use of high-performance computing (HPC) hardware to run a full optimization and would lead to extremely high runtime consumption. A single simulation of adequate resolution is executed in several minutes on modern HPC hardware, so that the total runtime of an optimization run would consume hours or even days to produce reasonable results. This was a fundamental restriction as to why an alternative ray-based approach was implemented.

From a computer science point of view, the optimization challenge we are facing here is essentially a real-valued optimization problem with independent continuous parameters. We are thus searching for optimal solutions in a huge solution space. Exhaustive search strategies are obviously not able to solve such problems in a reasonable time when based on non-linear physics solvers. Whereas genetic algorithms are known to produce great results for combinatorial optimization problems in feasible time, evolution strategies are perfectly designed to solve high-dimensional continuous optimization problems as they are population-based and are therefore able to scan huge search spaces efficiently, as well as due to their ability to initially perform diversification and later intensification of the search process by reducing the mutation depending on current optimization process dynamics. As we see in other projects [42,43], evolution strategies are able to solve highly complex optimization problems in various domains; whereas the main algorithmic idea may be very similar in all these approaches, the main difference and also the main challenge is how to define solution candidates, the phenotype (i.e., how the candidates are decoded), and the fitness function (i.e., how the quality of solutions is calculated on the basis of their phenotype).

In our research, the main key to success was the combination of an evolutionary self-adaptive optimization algorithm and a subsequent simulation-based results evaluation. One strand of future research we will pursue is the integration of machine learning-based surrogate models developed and validated on simulation results across a wide range of parameter settings. This will allow for improvements in the optimization process, eliminate the need for subsequent simulations, and directly increase the accuracy of the optimization algorithm. The large and diverse dataset required to accurately capture the complex, non-linear behavior of shockwaves during therapy is currently unavailable; this dataset will be generated through further research to enable the development of reliable surrogate models. Testing on one of the most challenging devices due to its stochastic signals and soft focus, we gained confidence in the optimization’s robustness in designing an effective reflector that remains robust to potential positional changes occurring during therapy sessions. Adapting the secondary reflector to devices with more well-defined foci and homogeneous signals can be achieved without changing the optimization process.

Modifying the reflector design to accommodate electrohydraulic devices with elliptical applicator heads is straightforward, as their well-defined focus simplifies the refocusing process. In contrast, electromagnetic devices, while still using parabolic reflectors, generate homogeneous signals, which reduces wave variability and simplifies positioning the secondary reflector as lateral misalignment is less of a concern. Finally, piezoelectric devices offer the simplest geometries for refocusing the initial shockwave. Their spherical, well-defined focal zones and homogeneous signals make them ideal for secondary reflector integration. Overall, the challenges addressed during the development of the reflector for electrohydraulic devices have already accounted for the most complex conditions encompassing the variations encountered in the other technologies.

This study has shown that naturally occurring regions of high-intensity tensile pressure that occur during traditional ESWT treatments of the penis can be successfully addressed and removed by introducing an IML on the far side of the application site. Introducing a matched secondary steel reflector to this setup enables the return and refocusing of the passing shockwaves and, thus, the re-introduction of a second shockwave pulse back into the treatment zone and a potentially increased treatment volume and number of pulses from a single applicator shot. In addition, the introduction of a secondary air reflector shows promising potential for further research applications in the study of tensile pressures and their role in ESWT regenerative ability, as it allows the controlled spatial separation and increase in focused tensile pressure exposure to the tissue.

## Figures and Tables

**Figure A1:**
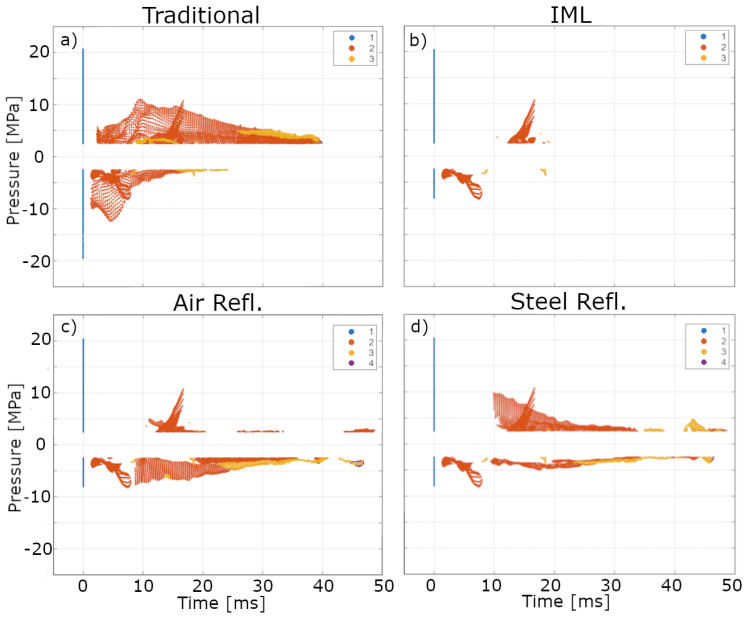
Scatter plots displaying the simulated peak pressure of a reflected shockwave pulse as a function of the time past the initial, at a given location for the four experimental configurations for a penis phantom with an irregular surface. The coloration indicates the enumeration of a subsequent peak, with the initial peaks seen in blue at time 0. Image (**a**) depicts the traditional setup, (**b**) includes the IML, (**c**) includes the IML and air reflector, and (**d**) shows the IML and steel reflector.

## Data Availability

The data presented in this study are available on request from the corresponding author. Some elements of the data may be considered proprietary.

## Abbreviations

The following abbreviations are used in this manuscript:

ED	Erectile dysfunction
IML	Impedance-matched layer
ES	Evolution Strategy
ESWT	Extracorporeal Shockwave Treatment
Li-ESWT	Low-Intensity Extracorporeal Shockwave Treatment
